# Supplementary material for: KidneyGenAfrica multi-cohort Genome-wide association study and polygenic prediction of kidney function in 110,000 Africans
Source: Nat Commun. 2026 Feb 10;17:2599. doi: 10.1038/s41467-026-69367-3 (PMC13003101; doi:10.1038/s41467-026-69367-3)
Supplement: Supplementary file 1 — Supplementary Information [file 41467_2026_69367_MOESM1_ESM.pdf]

KidneyGenAfrica multi-cohort Genome-wide association study and polygenic prediction of kidney function in 110,000 Africans

Abram B. Kamiza[1,2,3,4,5*], Tinashe Chikowore[6,7,8*], Guanjie Chen[9], Oyesola Ojewunmi[1,2], Tafadzwa Machipisa[10,11] ,Feng Zhou[1,12], Richard Mayanja[1,13], , Sounkou Toure[14], Opeyemi Soremekun[15,1], Christopher Kintu[1,2,16], Mariam Nakabuye[1,16,17], Mine Koprulu[2], Allan Kalungi[1,2], Robert Kalyesubula[16], Babatunde Salako[18],Oyekanmi Nashiru[19], Manuel Corpas[20,21], Cassianne Robinson-Cohen[22], Nora Franceschini[23], Cristian Pattaro[24], Anna Köttgen[25], Dorothea Nitsch[4], Claudia Langenberg[2,26], Catherine Tcheandjieu[27,28], Moffat Njirenda[1, 4], Andrew P Morris[29], Jennifer Asimit[12], Eleftheria Zeggini[15, 30], Charles Rotimi[9], Michele Ramsay[5], Adeyemo Adebowale[9], June Fabian[31,32], Amelia C. Crampin[3,33,34,35], Jean-Tristan Brandenburg[5,36], Segun Fatumo[1, 2, 4, #]

[1]Medical Research Council, Uganda Virus Research Institute and London School of Hygiene and Tropical Medicine (MRC/UVRI &LSHTM), Entebbe, Uganda; [2]Precision Healthcare University Research Institute, Queen Mary University of London; [3]Malawi Epidemiology and Intervention Research Unit, Lilongwe, Malawi; [4]Department of Non-Communicable Disease Epidemiology (NCDE), London School of Hygiene and Tropical Medicine, Keppel St, London, WC1E 7HT, UK; [5]Sydney Brenner Institute for Molecular Bioscience, Faculty of Health Sciences, University of the Witwatersrand, Johannesburg, South Africa.[6]MRC/Wits Developmental Pathways for Health Research Unit, Department of Paediatrics, Faculty of Health Sciences, University of the Witwatersrand, Johannesburg, South Africa; [7]Channing Division of Network Medicine, Department of Medicine, Brigham and Women's Hospital and Harvard Medical School, Boston, MA, USA; [8] Division of Genetics, Department of Medicine, Brigham and Women's Hospital and Harvard Medical School, Boston, MA, USA; [9]Center for Research on Genomics and Global Health, National Institute of Health, Bethesda, MD; [10]Department of Genetics, Perelman School of Medicine, University of Pennsylvania, Philadelphia, Pennsylvania, USA; [11]Hatter Institute for Cardiovascular Diseases Research in Africa (HICRA), Department of Medicine, University of Cape Town, Cape Town 7701, South Africa; [12]MRC Biostatistics Unit, University of Cambridge, Cambridge, UK; [13]Gladstone Institutes of Data Science and Biotechnology, Gladstone Institute, 1650 Owens street, San Francisco, CA, 94158, USA; [14]African Center of Excellence in Bioinformatics, University of Sciences, Techniques and Technologies of Bamako, Bamako, Mali; [15]Institute of Translational Genomics, Helmholtz Zentrum München – German Research Center for Environmental Health, 85764 Neuherberg, Germany; [16]Makerere University, Kampala, Uganda; [17]Copenhagen Health Complexity Center, Department of Public Health, University of Copenhagen, Denmark.[18]Nigerian Institute of Medical Research, Lagos, Nigeria; [19]Center for Genomics Research and Innovation, National Biotechnology Development Agency, Abuja, Nigeria; [20]College of Liberal Arts and Sciences, University of Westminster, London, UK; [21]Cambridge Precision Medicine Limited, ideaSpace, University of Cambridge Biomedical Innovation Hub, Cambridge, United Kingdom; [22]Division of Nephrology, Department of Medicine, Vanderbilt University Medical Center, Nashville, TN; [23]The University of North Carolina at Chapel Hill, Chapel Hill, North Carolina, United States; [24]Institute for Biomedicine, Eurac Research, Via Volta 21, 39100 Bolzano, Italy; [25]Institute of Genetic Epidemiology, Faculty of Medicine and Medical Center – University of Freiburg, Freiburg, Germany; [26]Berlin Institute of Health at Charité, Germany; [27]Gladstone Institutes of Data Science and Biotechnology, Gladstone Institute, 1650 Owens street, San Francisco, CA, 94158, USA; [28]Department of epidemiology and Biostatistics, University of California San Francisco, San Francisco, CA, USA; [29]Centre for Genetics and Genomics Versus Arthritis, University of Manchester, Manchester, UK; [30]TUM School of Medicine and Health, Technical University of Munich (TUM), TUM University Hospital, Munich, Germany; [31] Medical Research Council/Wits University Rural Public Health and Health Transitions Research Unit (Agincourt), School of Public Health, Faculty of Health Sciences, University of the Witwatersrand, Johannesburg, South Africa; [32]Wits Donald Gordon Medical Research Institute, Faculty of Health Sciences, University of the Witwatersrand, Johannesburg, South Africa; [33]School of Global and Public Health, Kamuzu University of Health Sciences; [34] School of Health and Wellbeing, University of Glasgow, Glasgow, UK; [35]Epidemiology and Population Health, London School of Hygiene and Tropical Medicine, London, UK. [36] Strengthening Oncology Services Research Unit, Faculty of Health Sciences, University of the Witwatersrand, Johannesburg, South Africa.

* These authors contributed equally

(a) ARK South Africa

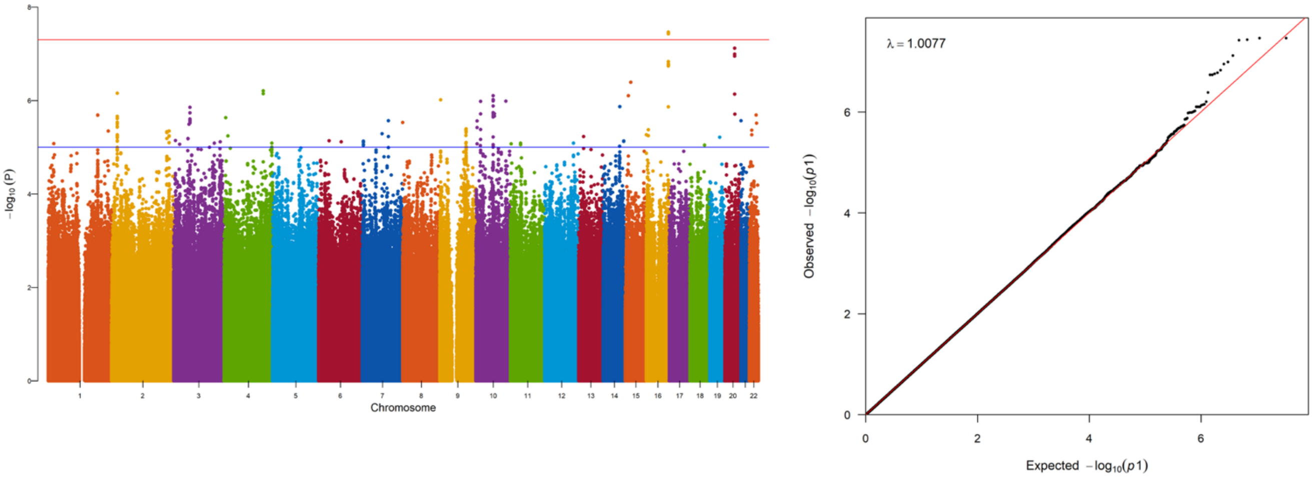

(b) MEIRU Malawi

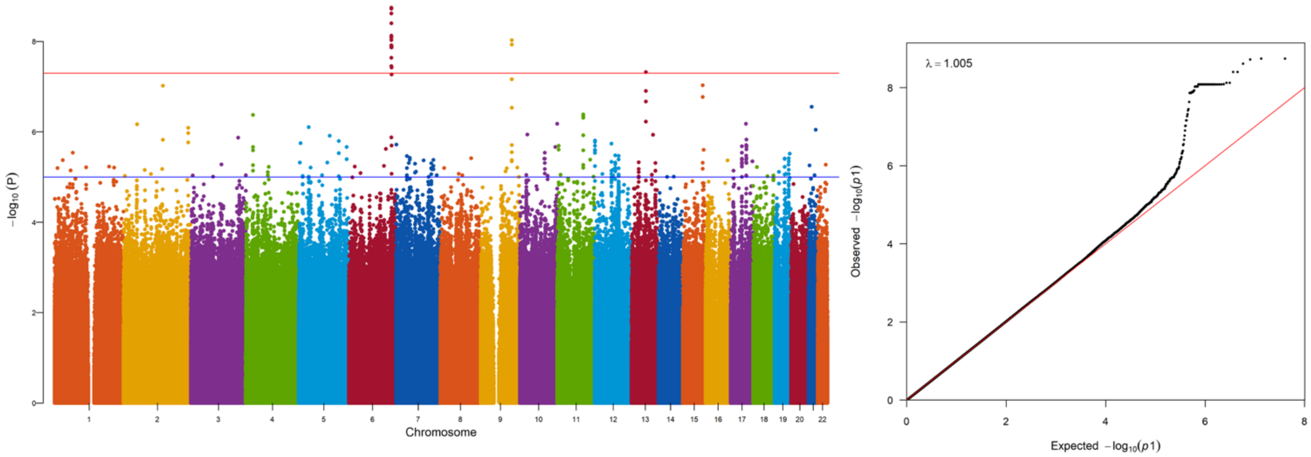

(c) AWI-Gen South Africa

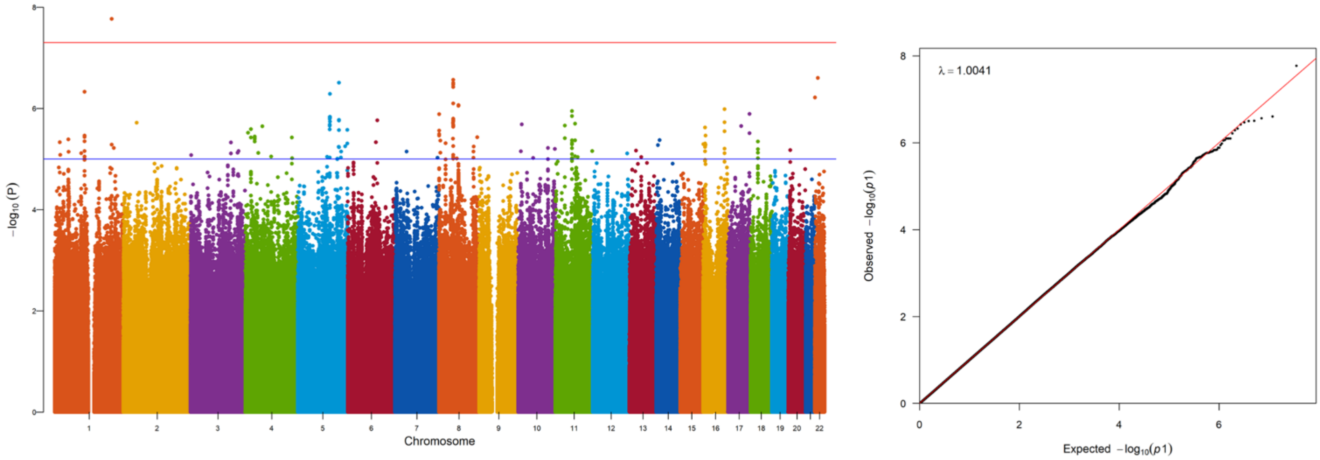

(d) UGR wave one Uganda

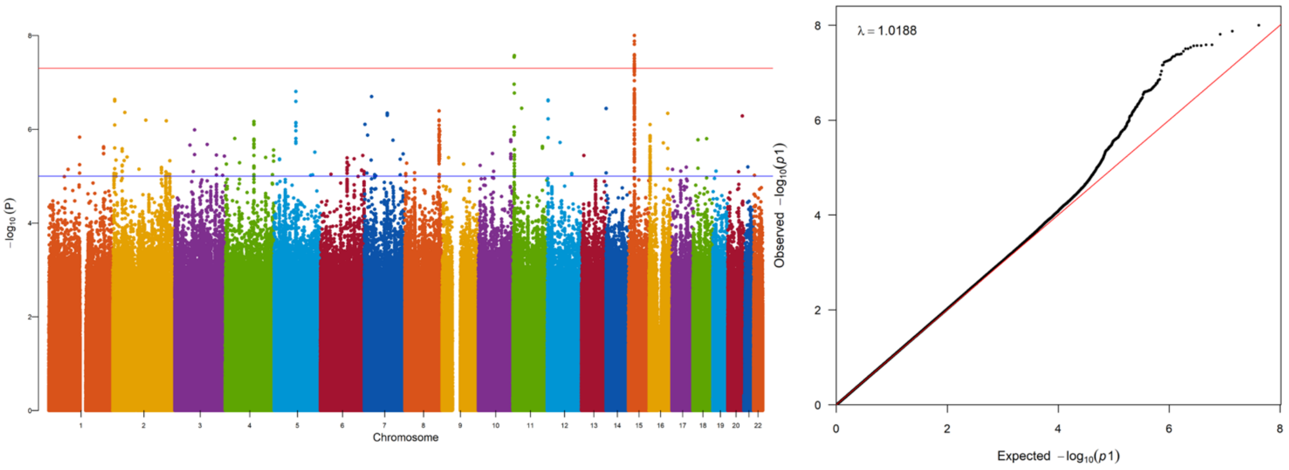

(e) AWI-Gen Kenya

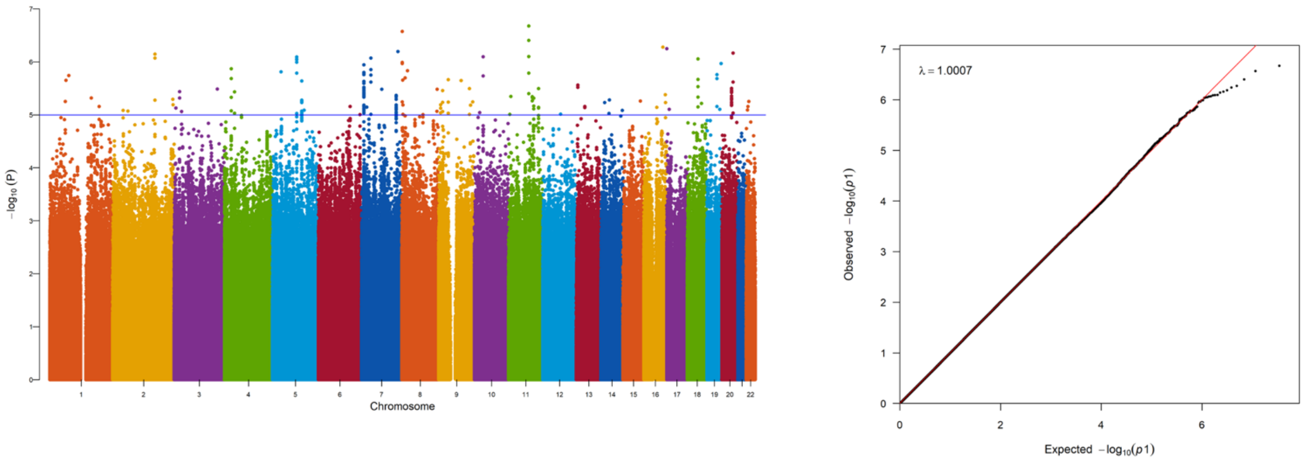

(f) AADM Kenya

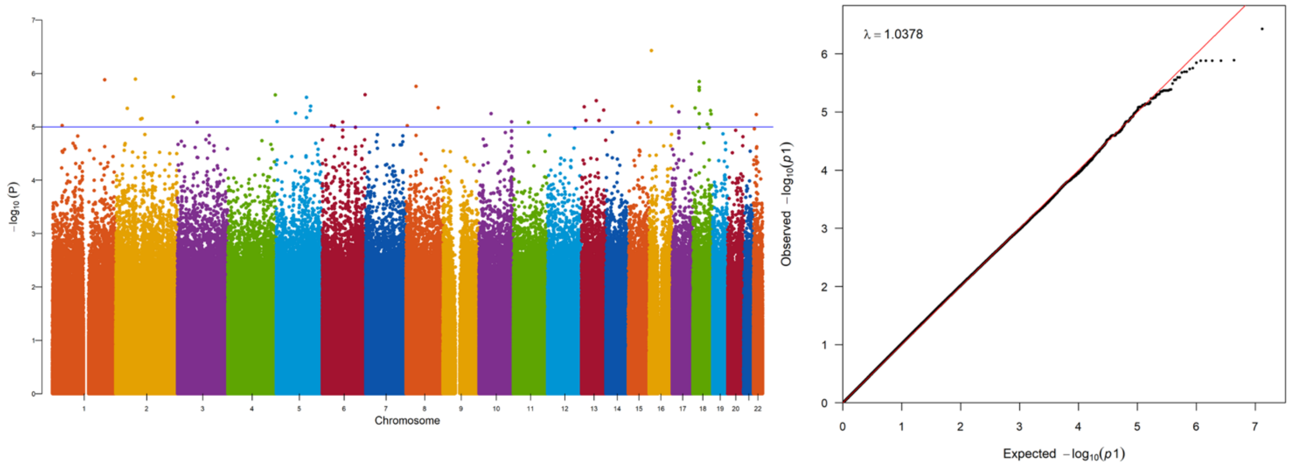

(g) AADM Ghana

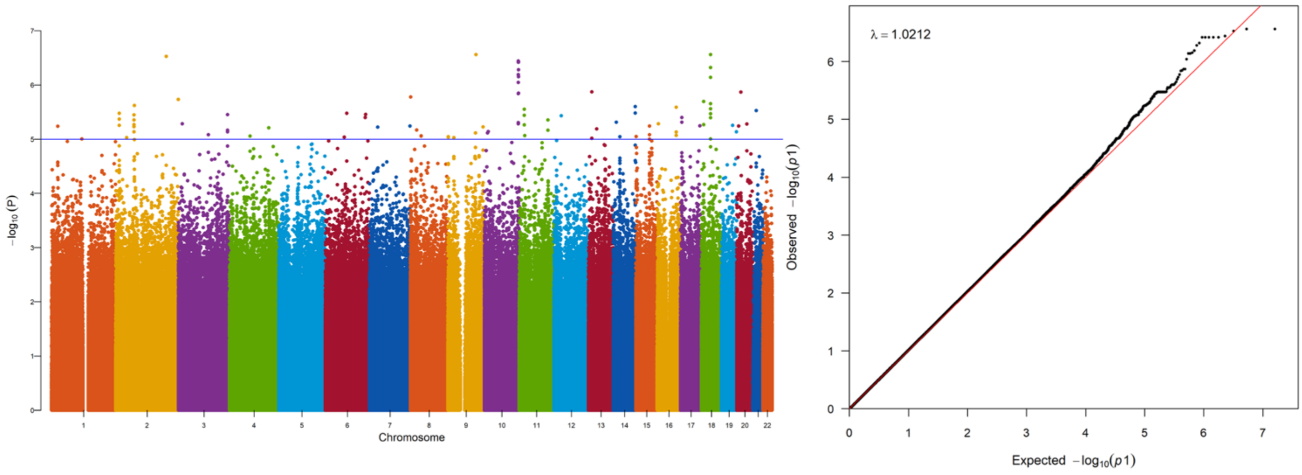

(h) AADM Nigeria

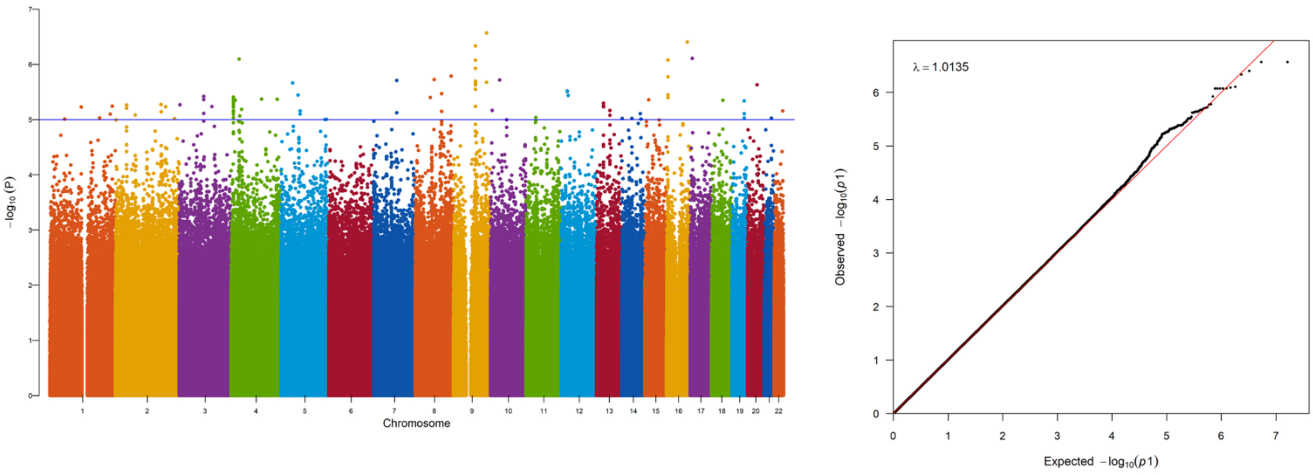

(i) AWI-Gen Burkina Faso and Ghana

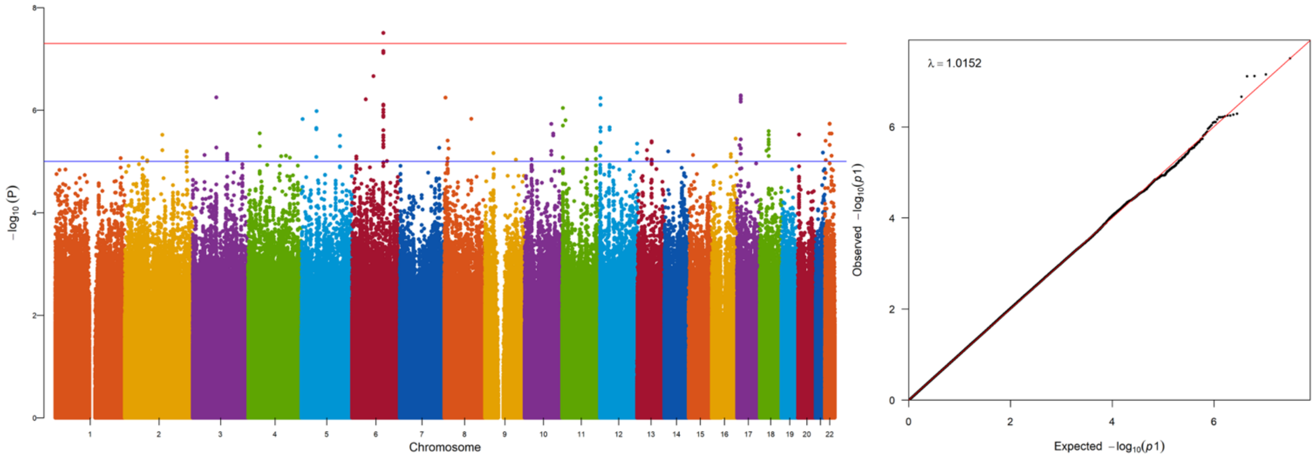

Supplementary Figure 1 Manhattan and QQ plot of contributing cohort and studies in KidneyGen, (a) ARK South Africa, (b) MEIRU Malawi, (c) AWI-Gen South Africa, (d) UGR Uganda, (e) AWI-Gen Kenya, (f) AADM Kenya, (g) AADM Ghana, (h) AADM Nigeria (i) AWI-Gen Burkina Faso and Ghana.

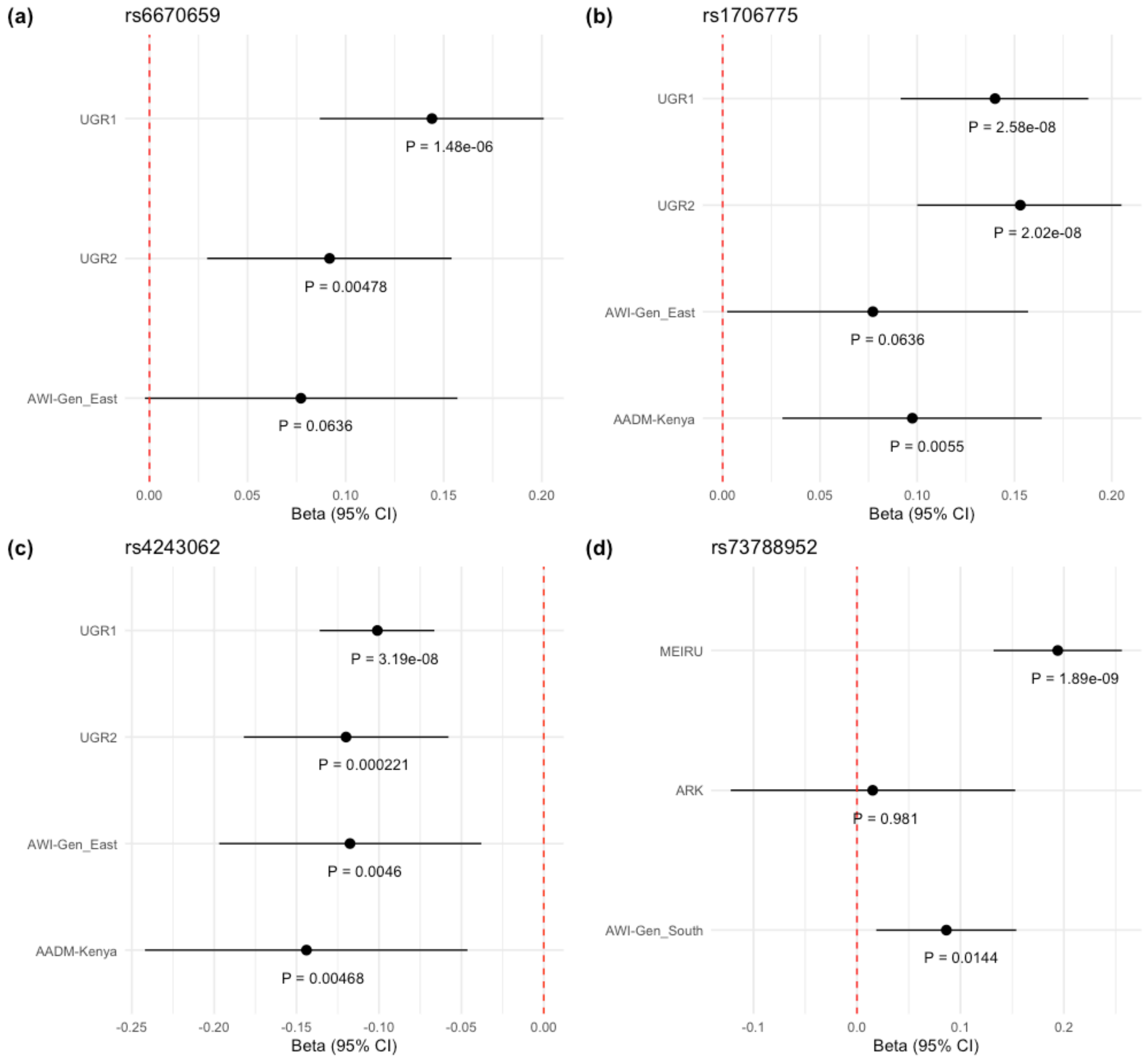

Supplementary Figure 2 Forest plot showing the effect size estimates (Beta) and 95% confidence intervals (CI) for the independent loci associated with eGFR. The horizontal lines represent 95% confidence intervals, and the black circles represent point estimates (Beta). P-values for each cohort are shown next to the respective estimates. The red dashed vertical line represents the null-effect line (Beta = 0). (a) **rs6670659** is an independent locus across three East African cohorts: Uganda Genome Resources (UGR) waves 1 and 2, and Africa Wits-INDEPTH partnership for Genomic Studies [AWI-Gen] East. (b) **rs1706775** is an independent locus across four East African cohorts: Uganda Genome Resources (UGR) waves 1 and 2, Africa America Diabetes (AADM) Kenya, and Africa Wits-INDEPTH partnership for Genomic Studies (AWI-Gen) East. (c) **rs4243062** is an independent novel locus across four East African cohorts: Uganda Genome Resources (UGR) waves 1 and 2, Africa America Diabetes (AADM), Kenya, and Africa Wits-INDEPTH partnership for Genomic Studies (AWI-Gen) East. (d) **rs73788952** is an independent novel locus across three Southern African cohorts: Malawi Epidemiology and Intervention Research Unit (MEIRU), African Research on Kidney Disease (ARK), and Africa Wits-INDEPTH partnership for Genomic Studies [AWI-Gen] South.

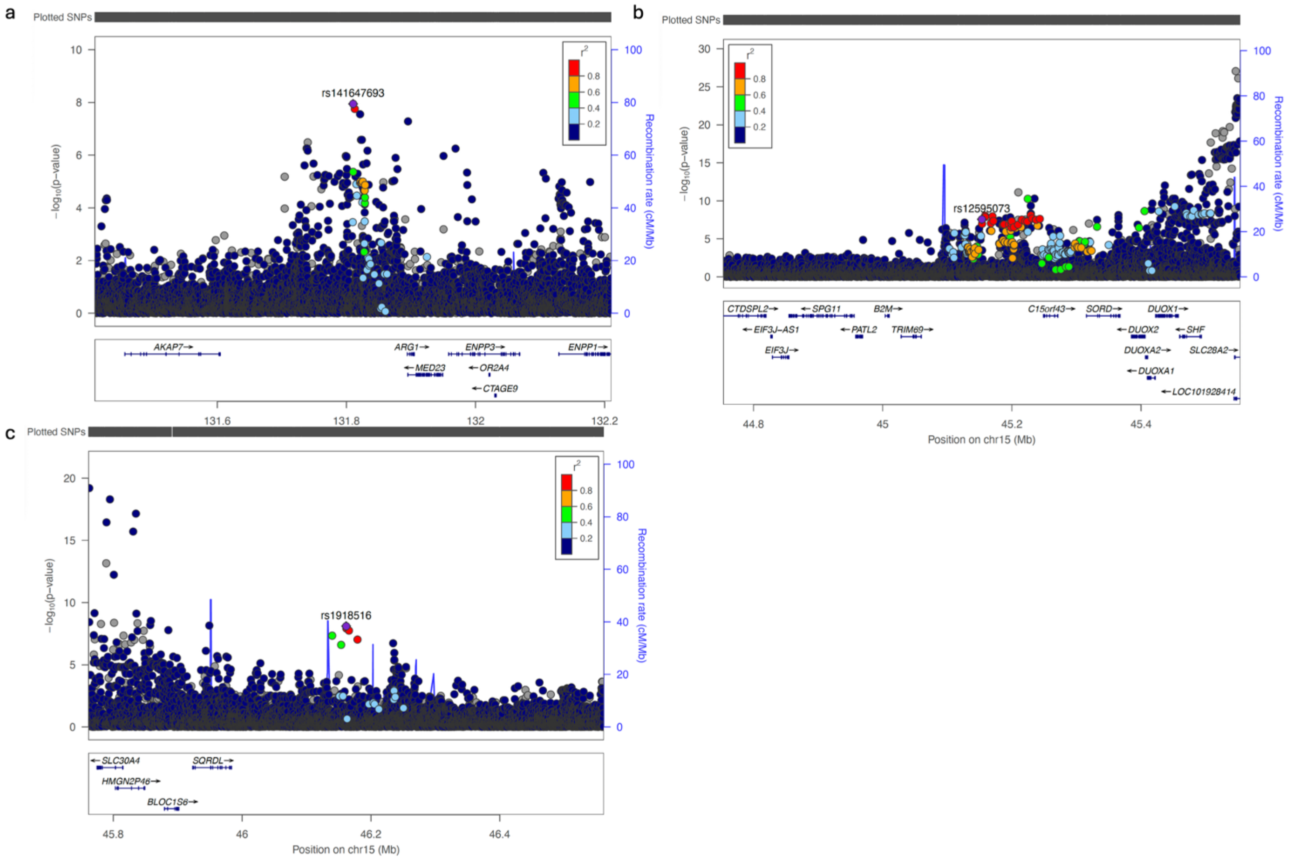

Supplementary Figure 3 Locus zoom plots for novel loci associated with eGFR in a pan-African meta-analysis of individuals of African ancestry. (a) rs141647693, (b) rs12595073, and (c) rs1918516 loci.

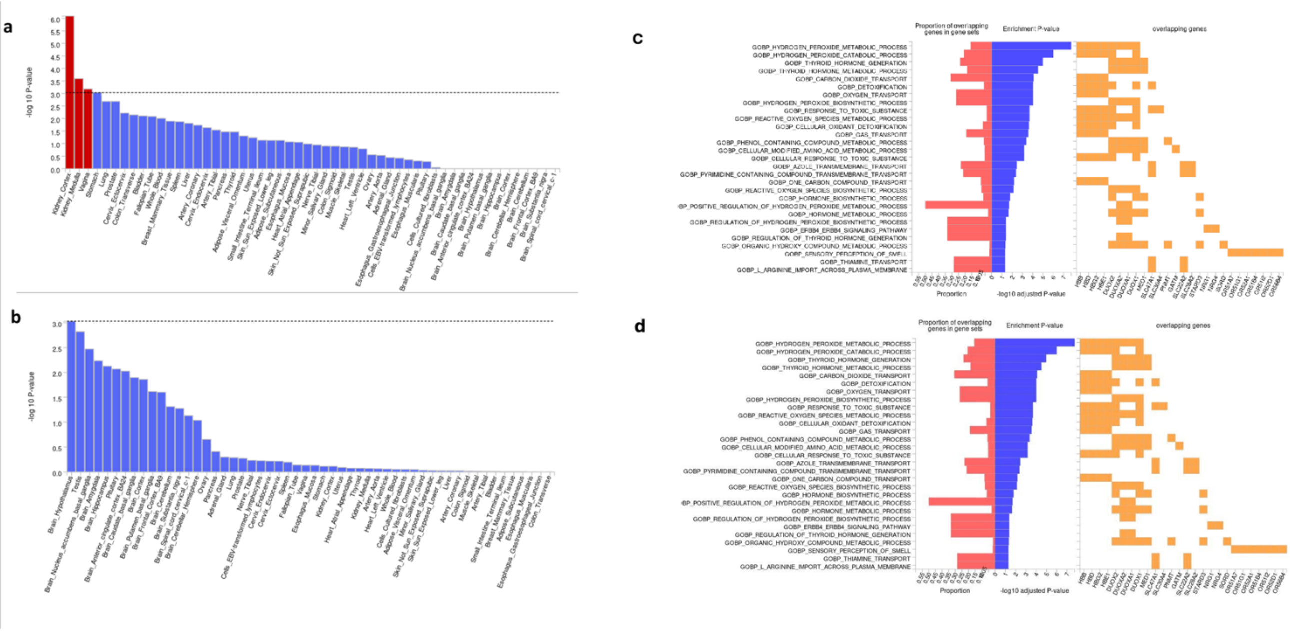

Supplementary Figure 4 Pathway analysis using gene-based analysis implemented in FUMA. Our results showed that the mapped genes from the combined African datasets were highly expressed in the kidney (cortex and medulla) and vagina.

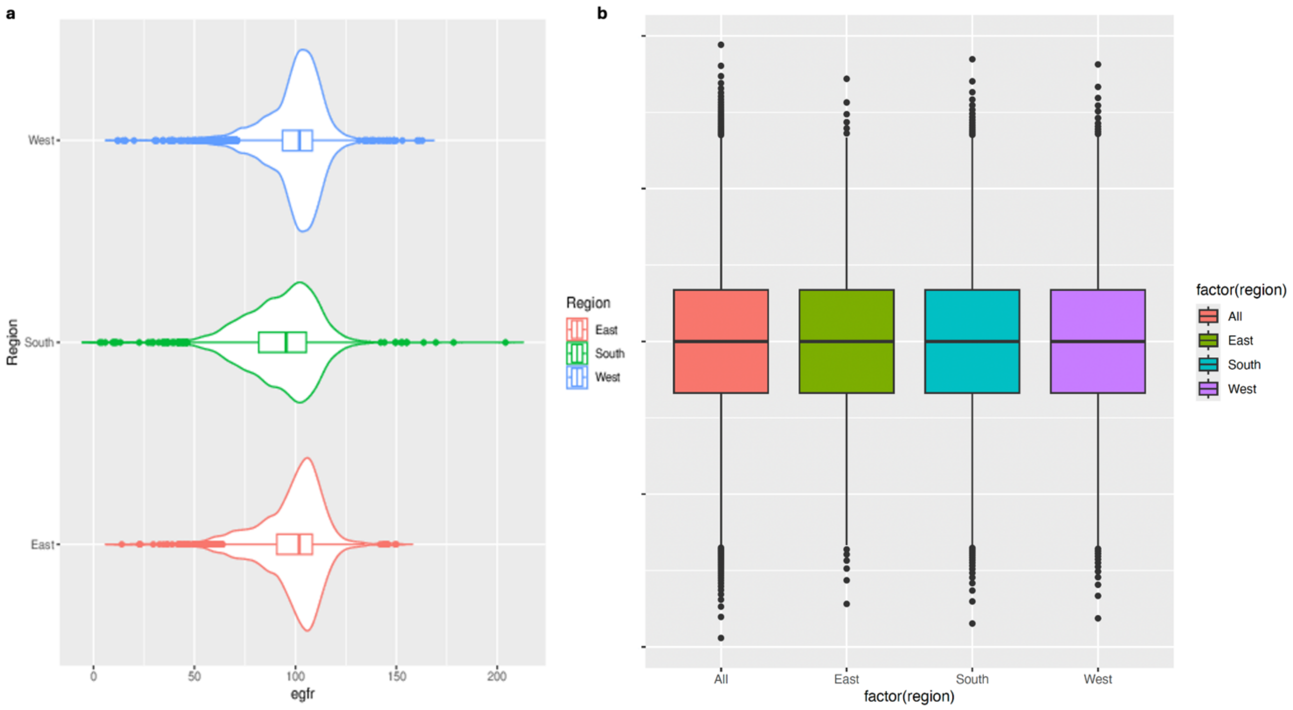

Supplementary Figure 5 Distribution of estimated glomerular filtration rate (eGFR) by region. (a) Violin plots with overlaid boxplots showing the distribution of eGFR in East, South and West regions. (b) Boxplots of eGFR after the inverse ranked transformation, for all participants and stratified by region. The horizontal lines indicate medians, and the interquartile ranges.

Supplementary Table 1 Basic characteristics of studies or cohorts included in this meta-analysis

Cohort/ study	Country of origin	Geographical Region	Sample (n (%)	Number of SNPs
MEIRU	Malawi	Southern Africa	6380 (5.83)	20,125,941
ARK	South Africa	Southern Africa	1060 (0.97)	16,709,600
AWI-Gen	South Africa	Southern Africa	4527 (4.14)	17,572,045
UGR wave 1	Uganda	Eastern Africa	4407 (4.01)	20,596,936
UGR wave 2	Uganda	Eastern Africa	2301(1.87)	20,596,936
AADM	Kenya	Eastern Africa	2069 (1.89)	6,518,420
AWI-Gen	Kenya	Eastern Africa	1704 (1.56)	17,265,628
AADM	Nigeria	Western Africa	2069 (1.89)	8,047,582
AADM	Ghana	Western Africa	1060 (0.97)	7,958,170
AWI-Gen	Burkina Faso & Ghana	Western Africa	3475 (3.18)	15,680,497
MVP	US	Diaspora Africa	57,336 (52.1)	16,427,567
UKB	UK	Diaspora Africa	6856 (6.27)	21,964,220
CKDGen	US, UK	Diaspora Africa	16,473 (15.06)	23,253,490

MEIRU; Malawi Epidemiology and Intervention Research Unit, ARK; African Research on Kidney Disease, AWI-Gen; Africa Wits-INDEPTH partnership for Genomic Studies, UGR; Uganda Genome Resources, AADM; Africa America Diabetes Mellitus, MVP; Million Veteran Program, UKB; UK BioBank; CKDGen; Chronic Kidney Disease Genetic, SNPs; single nucleotide polymorphisms

Supplementary Table 2 Independent loci associated with eGFR from contributing studies and cohorts in continental Africa

SNP	CHR	BP	EA	NEA	EAF	BETA	SE	P	N	Studies /cohort
rs74383679	1	206198189	G	C	0.161	0.175	0.0311	1.69e-08	4527	AWI-Gen South
rs114701867	6	154343357	A	G	0.108	0.173	2.87e-02	1.80e-09	6195	MEIRU
rs7763270	6	112537967	A	G	0.139	-0.193	0.0348	3.12e-08	3475	AWI-Gen West
rs10979975	9	112397085	G	A	0.094	0.176	3.066e-02	9.41e-09	6195	MEIRU
rs1223033645	11	5363496	G	A	0.935	0.294	5.278e-02	2.69e-08	3288	UGR1
rs115943222	13	70304786	A	C	0.009	-0.505	9.251e-02	4.79e-08	6195	MEIRU
rs2433603	15	45646226	T	C	0.481	0.144	2.517e-02	1.01e-08	3288	UGR1
rs8039921	15	45582237	A	G	0.498	0.162	2.724e-02	2.55e-09	2773	UGR2
rs7189561	16	86714129	A	T	0.02	-0.912	0.163	3.44e-08	965.0	ARK

SNP; single nucleotide polymorphism, CHR chromosome, BP, base pair position, EA; effect allele, NEA, non-effect allele, EAF; Effect allele frequency, SE; standard error

Supplementary Table 3: Heterogeneity metrics of lead single nucleotide polymorphisms associated with eGFR from regional meta-analysis

SNP	EA	NEA	EAF	BETA	SE	p-value	Q_statistic	Q_p-value	I2	Studies	Samples	Effects	Region
rs6670659	C	G	0.769	0.110	0.019	1.24E-08	2.229	0.328	0.102	3	7765	+++?	East
rs1706775	T	C	0.572	0.133	0.015	3.85E-17	1.749	0.625	0.000	4	9834	++++	East
rs4243062*	T	C	0.299	-0.100	0.018	3.19E-08	3.027	0.387	0.008	4	9834	----	East
rs73788952*	G	A	0.092	0.132	0.022	5.04E-09	8.001	0.018	0.750	3	11687	+++	South

SNP; single nucleotide polymorphism, EA; effect allele, NEA; non-effect allele, EAF; effect allele frequency, SE; standard error, Studies; number of participating cohorts or studies from a region, Sample; number of individual from studies used in meta-analysis, **+**; BETA estimate was positively associated with eGFR, **-**; BETA estimate was negatively associated with eGFR, **?**; SNPs not available in participating cohort or studies; *novel association

Supplementary Table 4 Phenome-wide association study in performed in pan-Africa ancestry

atlas ID	PMID	Year	Domain	Trait	P-value
4205	31152163	2019	Metabolic	Estimated glomerular filtration rate	1.51E-131
4206	31152163	2019	Metabolic	Estimated glomerular filtration rate	1.10E-124
4215	31015462	2019	Metabolic	Estimated glomerular filtration rate	3.31E-53
4063	28452372	2017	Metabolic	Estimated glomerular filtration rate based on serum creatinine	2.80E-39
862	27005778	2016	Metabolic	Creatinine	1.38E-13
4210	31152163	2019	Metabolic	Chronic kidney disease	2.77E-13
4209	31152163	2019	Metabolic	Chronic kidney disease	7.54E-13
3447	31427789	2019	Metabolic	Impedance measures - Impedance of whole body	1.25E-09
4357	30220432	2018	Metabolic	Albuminuria	3.65E-09
3470	31427789	2019	Metabolic	Impedance measures - Trunk fat-free mass	1.02E-08
3471	31427789	2019	Metabolic	Impedance measures - Trunk predicted mass	1.18E-08
3451	31427789	2019	Metabolic	Impedance measures - Impedance of arm (left)	1.68E-07
3444	31427789	2019	Metabolic	Impedance measures - Whole body water mass	4.81E-07
3466	31427789	2019	Metabolic	Impedance measures - Arm fat-free mass (left)	6.48E-07
4204	31217584	2019	Metabolic	Estimated glomerular filtration rate	6.53E-07
3443	31427789	2019	Metabolic	Impedance measures - Whole body fat-free mass	1.125E-06
3467	31427789	2019	Metabolic	Impedance measures - Arm predicted mass (left)	1.214E-06
3450	31427789	2019	Metabolic	Impedance measures - Impedance of arm (right)	2.364E-06
3462	31427789	2019	Metabolic	Impedance measures - Arm fat-free mass (right)	2.857E-06
3463	31427789	2019	Metabolic	Impedance measures - Arm predicted mass (right)	2.944E-06
3449	31427789	2019	Metabolic	Impedance measures - Impedance of leg (left)	6.054E-06
3448	31427789	2019	Metabolic	Impedance measures - Impedance of leg (right)	9.513E-06
3446	31427789	2019	Metabolic	Impedance measures - Basal metabolic rate	0.00001776
4301	30664634	2019	Metabolic	Arms-arm fat ratio (male)	0.00003188
4109	29403010	2018	Metabolic	Serum creatinine	0.0001925

Supplementary Table 5 Distribution of APOL1 gene in continental Africa

Haplotypes	east AFR<br>N = 8075<br>n (%)	south AFR<br>N = 11743<br>n (%)	west AFR<br>N = 3473<br>n (%)	All<br>N = 23291<br>n (%)
G0/G0	5908 (73.2)	6386 (54.4)	2082 (59.9)	14376 (61.7)
G0/G1	957 (11.9)	1715 (14.6)	699 (20.1)	3371 (14.5)
G0/G2	1035 (12.8)	2778 (23.7)	546 (15.7)	4359 (18.7)
G1/G1	30 (0.4)	94 (0.8)	34 (1)	158 (0.7)
G1/G2	89 (1.1)	420 (3.6)	77 (2.2)	586 (2.5)
G2/G2	56 (0.7)	350 (2.9)	35 (1)	441 (1.9)
High-risk haplotypes	175 (2.2)	864 (7.4)	146 (4.2)	1185 (5.1)
Alleles				
G0 allele	13808 (85.5)	17265 (73.5)	5409 (77.9)	36482 (78.3)
G1 allele	1106 (6.8)	2323 (9.9)	844 (12.2)	4273 (9.2)
G2 allele	1236 (7.7)	3898 (16.6)	693 (10)	5827 (12.5)
