# Supplementary material for: KidneyGenAfrica multi-cohort Genome-wide association study and polygenic prediction of kidney function in 110,000 Africans
Source: Nat Commun. 2026 Feb 10;17:2599. doi: 10.1038/s41467-026-69367-3 (PMC13003101; doi:10.1038/s41467-026-69367-3)
Supplement: Supplementary file 2 — Description of Additional Supplementary Files [file 41467_2026_69367_MOESM2_ESM.pdf]

Description of Supplementary Data

KidneyGenAfrica multi-cohort genome-wide association study and polygenic prediction of kidney function in 110,000 Africans

Abram B. Kamiza[1,2,3,4,5*], Tinashe Chikowore[6,7,8]*, Guanjie Chen[9], Oyesola Ojewunmi[1,2], Tafadzwa Machipisa[10,11] ,Feng Zhou[1,12], Richard Mayanja[1,13], , Sounkou Toure[14], Opeyemi Soremekun[15,1], Christopher Kintu[1,2,16], Mariam Nakabuye[1,16,17], Mine Koprulu[2], Allan Kalungi[1,2], Robert Kalyesubula[16], Babatunde Salako[18],Oyekanmi Nashiru[19], Manuel Corpas[20,21], Cassianne Robinson-Cohen[22], Nora Franceschini[23], Cristian Pattaro[24], Anna Köttgen[25], Dorothea Nitsch[4], Claudia Langenberg[2,26], Catherine Tcheandjieu[27,28], Moffat Njirenda[1, 4], Andrew P Morris[29], Jennifer Asimit[12], Eleftheria Zeggini[15, 30], Charles Rotimi[9], Michele Ramsay[5], Adeyemo Adebowale[9], June Fabian[31,32], Amelia C. Crampin[3,33,34,35], Jean-Tristan Brandenburg[5,36], Segun Fatumo[1, 2, 4, #]

[1]Medical Research Council, Uganda Virus Research Institute and London School of Hygiene and Tropical Medicine (MRC/UVRI &LSHTM), Entebbe, Uganda; [2]Precision Healthcare University Research Institute, Queen Mary University of London; [3]Malawi Epidemiology and Intervention Research Unit, Lilongwe, Malawi; [4]Department of Non-Communicable Disease Epidemiology (NCDE), London School of Hygiene and Tropical Medicine, Keppel St, London, WC1E 7HT, UK; [5]Sydney Brenner Institute for Molecular Bioscience, Faculty of Health Sciences, University of the Witwatersrand, Johannesburg, South Africa.[6]MRC/Wits Developmental Pathways for Health Research Unit, Department of Paediatrics, Faculty of Health Sciences, University of the Witwatersrand, Johannesburg, South Africa; [7]Channing Division of Network Medicine, Department of Medicine, Brigham and Women's Hospital and Harvard Medical School, Boston, MA, USA; [8] Division of Genetics, Department of Medicine, Brigham and Women's Hospital and Harvard Medical School, Boston, MA, USA; [9]Center for Research on Genomics and Global Health, National Institute of Health, Bethesda, MD; [10]Department of Genetics, Perelman School of Medicine, University of Pennsylvania, Philadelphia, Pennsylvania, USA; [11]Hatter Institute for Cardiovascular Diseases Research in Africa (HICRA), Department of Medicine, University of Cape Town, Cape Town 7701, South Africa; [12]MRC Biostatistics Unit, University of Cambridge, Cambridge, UK; [13]Gladstone Institutes of Data Science and Biotechnology, Gladstone Institute, 1650 Owens street, San Francisco, CA, 94158, USA; [14]African Center of Excellence in Bioinformatics, University of Sciences, Techniques and Technologies of Bamako, Bamako, Mali; [15]Institute of Translational Genomics, Helmholtz Zentrum München – German Research Center for Environmental Health, 85764 Neuherberg, Germany; [16]Makerere University, Kampala, Uganda; [17]Copenhagen Health Complexity Center, Department of Public Health, University of Copenhagen, Denmark.[18]Nigerian Institute of Medical Research, Lagos, Nigeria; [19]Center for Genomics Research and Innovation, National Biotechnology Development Agency, Abuja, Nigeria; [20]College of Liberal Arts and Sciences, University of Westminster, London, UK; [21]Cambridge Precision Medicine Limited, ideaSpace, University of Cambridge Biomedical Innovation Hub, Cambridge, United Kingdom; [22]Division of Nephrology, Department of Medicine, Vanderbilt University Medical Center, Nashville, TN; [23]The University of North Carolina at Chapel Hill, Chapel Hill, North Carolina, United States; [24]Institute for Biomedicine, Eurac Research, Via Volta 21, 39100 Bolzano, Italy; [25]Institute of Genetic Epidemiology, Faculty of Medicine and Medical Center – University of Freiburg, Freiburg, Germany; [26]Berlin Institute of Health at Charité, Germany; [27]Gladstone Institutes of Data Science and Biotechnology, Gladstone Institute, 1650 Owens street, San Francisco, CA, 94158, USA; [28]Department of epidemiology and Biostatistics, University of California San Francisco, San Francisco, CA, USA; [29]Centre for Genetics and Genomics Versus Arthritis, University of Manchester, Manchester, UK; [30]TUM School of Medicine and Health, Technical University of Munich (TUM), TUM University Hospital, Munich, Germany; [31] Medical Research Council/Wits University Rural Public Health and Health Transitions Research Unit (Agincourt), School of Public Health, Faculty of Health Sciences, University of the Witwatersrand, Johannesburg, South Africa; [32]Wits Donald Gordon Medical Research Institute, Faculty of Health Sciences, University of the Witwatersrand, Johannesburg, South Africa; [33]School of Global and Public Health, Kamuzu University of Health Sciences; [34] School of Health and Wellbeing, University of Glasgow, Glasgow, UK; [35]Epidemiology and Population Health, London School of Hygiene and Tropical Medicine, London, UK. [36] Strengthening Oncology Services Research Unit, Faculty of Health Sciences, University of the Witwatersrand, Johannesburg, South Africa.

* These authors contributed equally

Supplementary Data 1 Lack of regional replication of lead single nucleotide polymorphisms in the three geographical regions in Africa

Supplementary Data 2 Replication of lead single nucleotide polymorphisms within the regional meta-analyses

Supplementary Data 3 Fine mapping of eGFR in the pan-African meta-analysis

Supplementary Data 4 Colocalization in pan-African meta-analysis

Supplementary Data 5-8 FUMA results

Supplementary Data 9 Parameters used for identifying best performing polygenic scores for replication
