# Supplementary material for: KidneyGenAfrica multi-cohort Genome-wide association study and polygenic prediction of kidney function in 110,000 Africans
Source: Nat Commun. 2026 Feb 10;17:2599. doi: 10.1038/s41467-026-69367-3 (PMC13003101; doi:10.1038/s41467-026-69367-3)
Supplement: Supplementary file 9 — Reporting Summary [file 41467_2026_69367_MOESM9_ESM.pdf]

nature portfolio

Corresponding author(s): Segun Fatumo

Last updated by author(s): Jun 10, 2025

Reporting Summary

Nature Portfolio wishes to improve the reproducibility of the work that we publish. This form provides structure for consistency and transparency in reporting. For further information on Nature Portfolio policies, see our Editorial Policies and the Editorial Policy Checklist.

Statistics

For all statistical analyses, confirm that the following items are present in the figure legend, table legend, main text, or Methods section.

n/a	Confirmed	
☐	☒	The exact sample size (*n*) for each experimental group/condition, given as a discrete number and unit of measurement
☒	☐	A statement on whether measurements were taken from distinct samples or whether the same sample was measured repeatedly
☐	☒	The statistical test(s) used AND whether they are one- or two-sided<br>*Only common tests should be described solely by name; describe more complex techniques in the Methods section.*
☐	☒	A description of all covariates tested
☐	☒	A description of any assumptions or corrections, such as tests of normality and adjustment for multiple comparisons
☐	☒	A full description of the statistical parameters including central tendency (e.g. means) or other basic estimates (e.g. regression coefficient) AND variation (e.g. standard deviation) or associated estimates of uncertainty (e.g. confidence intervals)
☐	☒	For null hypothesis testing, the test statistic (e.g. *F*, *t*, *r*) with confidence intervals, effect sizes, degrees of freedom and *P* value noted<br>*Give P values as exact values whenever suitable.*
☐	☒	For Bayesian analysis, information on the choice of priors and Markov chain Monte Carlo settings
☐	☒	For hierarchical and complex designs, identification of the appropriate level for tests and full reporting of outcomes
☐	☒	Estimates of effect sizes (e.g. Cohen's *d*, Pearson's *r*), indicating how they were calculated

Our web collection on statistics for biologists contains articles on many of the points above.

Software and code

Policy information about availability of computer code

Data collection	No software was used for data collection
Data analysis	easyQC, Crossmap, GEMMA, GWAMA , Metasoft, METAL, JAM, coloc, LocusZoom, FUMA (https://fuma.ctglab.nl/), PheWAS, PRSice-2

For manuscripts utilizing custom algorithms or software that are central to the research but not yet described in published literature, software must be made available to editors and reviewers. We strongly encourage code deposition in a community repository (e.g. GitHub). See the Nature Portfolio guidelines for submitting code & software for further information.

Data

Policy information about availability of data

All manuscripts must include a data availability statement. This statement should provide the following information, where applicable:

- Accession codes, unique identifiers, or web links for publicly available datasets
- A description of any restrictions on data availability
- For clinical datasets or third party data, please ensure that the statement adheres to our policy

The genome-wide association study summary statistic data generated in this study have been deposited in the GWAS Catalog database under accession code [add hyperlink here].

Research involving human participants, their data, or biological material

Policy information about studies with human participants or human data. See also policy information about sex, gender (identity/presentation), and sexual orientation and race, ethnicity and racism.

Reporting on sex and gender	All GWAS summary data utilized in this analysis contained information on self-reported sex. Nonetheless, we did not conduct a sex-stratified analysis.
Reporting on race, ethnicity, or other socially relevant groupings	The study is reporting findings in individuals of African ancestry in the US, UK, and continental Africa.
Population characteristics	The majority of the participants included in the study were between the ages of 30-60 and comprised both males and females. Along with self-reported age and sex, the study also collected the identity of the participants, as well as samples for creatinine measurement and genotyping. The characteristics of the participants, including covariates, are included in the supplementary data.
Recruitment	We included individuals of African ancestry from eastern Africa (Africa Wits-INDEPTH partnership for Genomic Studies [AWI-Gen] Kenya, Uganda Genome Resources [UGR], Uganda, and Africa America Diabetes (AADM) study, Kenya), western Africa (AWI-Gen Ghana, AWI-Gen-Burkina Fatso, AADM-Ghana, and AADM-Nigeria), and southern Africa (Malawi Epidemiology and Interversion Research Unit [MEIRU], Malawi, AWi-Gen-South Africa, and African Research on Kidney Disease [ARK] study. We also inluded summary data from the Million Veteran Program (MVP), UK Biobank (UKBB), and Chronic Kidney Disease Genetic Consortium (CKD-Gen) in individuals of African ancestry.
Ethics oversight	All studies included in this analysis were approved by the individual cohort, respectively.

Note that full information on the approval of the study protocol must also be provided in the manuscript.

Field-specific reporting

Please select the one below that is the best fit for your research. If you are not sure, read the appropriate sections before making your selection.

☒ Life sciences ☐ Behavioural & social sciences ☐ Ecological, evolutionary & environmental sciences

For a reference copy of the document with all sections, see nature.com/documents/nr-reporting-summary-flat.pdf

Life sciences study design

All studies must disclose on these points even when the disclosure is negative.

Sample size	Genotyped data of 110,000 individuals of African ancestry were analysed in this study. These individuals are from MEIRU(n=6380), ARK(n=1060), AWI-Gen-South Afria (n=4527), UGR wave 1(n=4407), UGR wave 2(n=2301), AADM-Kenya (n=2069), AWI-Gen-Kenya (n=1704), AADM-Nigeria (n=2069), AADM-Ghana (n=1060), AWI-Gen-Burkina Faso and Ghana (n=3475), MVP(n=57336), UKBB(n=6856), CKDGen(n=16473)
Data exclusions	We excluded individuals and single nucleotide polymorphisms failing the stringent quality control procedures.
Replication	The replication was carried out by observing whether the same signal identified in our study was observed in other GWAS cohorts.
Randomization	We did not allocate any samples to groups, hence randomization was not relevant in this study.
Blinding	The study does not involve treatment, hence blinding was not relevant.

Reporting for specific materials, systems and methods

We require information from authors about some types of materials, experimental systems and methods used in many studies. Here, indicate whether each material, system or method listed is relevant to your study. If you are not sure if a list item applies to your research, read the appropriate section before selecting a response.

Materials & experimental systems

n/a	Involved in the study
☒	☐ Antibodies
☒	☐ Eukaryotic cell lines
☒	☐ Palaeontology and archaeology
☒	☐ Animals and other organisms
☒	☐ Clinical data
☒	☐ Dual use research of concern
☒	☐ Plants

Methods

n/a	Involved in the study
☒	☐ ChIP-seq
☒	☐ Flow cytometry
☒	☐ MRI-based neuroimaging

Plants

Seed stocks

Report on the source of all seed stocks or other plant material used. If applicable, state the seed stock centre and catalogue number. If plant specimens were collected from the field, describe the collection location, date and sampling procedures.

Novel plant genotypes

Describe the methods by which all novel plant genotypes were produced. This includes those generated by transgenic approaches, gene editing, chemical/radiation-based mutagenesis and hybridization. For transgenic lines, describe the transformation method, the number of independent lines analyzed and the generation upon which experiments were performed. For gene-edited lines, describe the editor used, the endogenous sequence targeted for editing, the targeting guide RNA sequence (if applicable) and how the editor was applied.

Authentication

Describe any authentication procedures for each seed stock used or novel genotype generated. Describe any experiments used to assess the effect of a mutation and, where applicable, how potential secondary effects (e.g. second site T-DNA insertions, mosiacism, off-target gene editing) were examined.
